# Early care and support for young children with developmental disabilities and their caregivers in Uganda: The Baby Ubuntu feasibility trial

**DOI:** 10.3389/fped.2022.981976

**Published:** 2022-09-13

**Authors:** Carol Nanyunja, Samantha Sadoo, Maya Kohli-Lynch, Ruth Nalugya, James Nyonyintono, Anita Muhumuza, Kenneth R. Katumba, Emily Trautner, Brooke Magnusson, Daniel Kabugo, Frances M. Cowan, Maria Zuurmond, Catherine Morgan, Deborah Lester, Janet Seeley, Emily L. Webb, Christine Otai, Giulia Greco, Margaret Nampijja, Cally J. Tann

**Affiliations:** ^1^MRC/UVRI and LSHTM Uganda Research Unit, Entebbe, Uganda; ^2^London School of Hygiene & Tropical Medicine, London, United Kingdom; ^3^Centre for Academic Child Health, Population Health Sciences, University of Bristol, Bristol, United Kingdom; ^4^Spina Bifida and Hydrocephalus Association of Uganda (SHA-U), Mbale, Uganda; ^5^Kiwoko Hospital, Nakaseke, Uganda; ^6^Adara Development, Edmonds, WA, United States; ^7^Mulago National Referral Hospital, Kampala, Uganda; ^8^Department of Paediatrics, Imperial College London, London, United Kingdom; ^9^Cerebral Palsy Alliance Research Institute, Specialty of Child and Adolescent Health, Sydney Medical School, Faculty of Medicine and Health, The University of Sydney, Sydney, NSW, Australia; ^10^Canadian Red Cross, Victoria, BC, Canada; ^11^African Population and Health Research Center, Nairobi, Kenya; ^12^Neonatal Medicine, University College London Hospitals NHS Foundation Trust, London, United Kingdom

**Keywords:** parenting program, early intervention, developmental disability, feasibility trial, caregiver, young children, Uganda

## Abstract

**Background:**

Early care and support provision for young children with developmental disabilities is frequently lacking, yet has potential to improve child and family outcomes, and is crucial for promoting access to healthcare and early education. We evaluated the feasibility, acceptability, early evidence of impact and provider costs of the Baby Ubuntu participatory, peer-facilitated, group program for young children with developmental disabilities and their caregivers in Uganda.

**Materials and methods:**

A feasibility trial, with two parallel groups, compared Baby Ubuntu with standard care. Caregivers and children, aged 6–11 months with moderate-severe neurodevelopmental impairment, were recruited and followed for 12 months. Quantitative and qualitative methods captured information on feasibility (ability to recruit), acceptability (satisfactory attendance), preliminary evidence of impact (family quality of life) and provider costs.

**Results:**

One hundred twenty-six infants (median developmental quotient, 28.7) were recruited and randomized (63 per arm) over 9 months, demonstrating feasibility; 101 (80%) completed the 12-month follow-up assessment (9 died, 12 were lost to follow up, 4 withdrew). Of 63 randomized to the intervention, 59 survived (93%); of these, 51 (86%) attended ≥6 modules meeting acceptability criteria, and 49 (83%) completed the 12 month follow-up assessment. Qualitatively, Baby Ubuntu was feasible and acceptable to caregivers and facilitators. Enabling factors included community sensitization by local champions, positive and caring attitudes of facilitators toward children with disability, peer support, and the participatory approach to learning. Among 101 (86%) surviving children seen at 12 months, mixed methods evaluation provided qualitative evidence of impact on family knowledge, skills, and attitudes, however impact on a scored family quality of life tool was inconclusive. Barriers included stigma and exclusion, poverty, and the need to manage expectations around the child’s progress. Total provider cost for delivering the program per participant was USD 232.

**Conclusion:**

A pilot feasibility trial of the Baby Ubuntu program found it to be feasible and acceptable to children, caregivers and healthcare workers in Uganda. A mixed methods evaluation provided rich programmatic learning including qualitative, but not quantitative, evidence of impact. The cost estimate represents a feasible intervention for this vulnerable group, encouraging financial sustainability at scale.

**Clinical trial registration:**

[https://doi.org/10.1186/ISRCTN44380971], identifier [ISRCTN44380971].

## Introduction

Addressing the needs of the 53 million children under 5 years of age living with developmental disabilities is a global priority (1), with early child development, inclusive of early childhood disability, recognized in the current Sustainable Development Goal (SDG) era. This has been strongly supported by the United Nations Global Strategy for Women’s, Children’s and Adolescents’ Health advocating for all children to not only “survive” but also “thrive” through community and service transformation (2). Supporting young children with disabilities and their caregivers to access inclusive healthcare and early education, remains a crucial component of the SDGs.

Child survivors of common neonatal conditions, such as neonatal encephalopathy (newborn brain injury), preterm birth and neonatal infections, are “at risk” of a wide spectrum of neurodevelopmental difficulties, delays and disabilities (3). These include cerebral palsy and other global developmental disabilities which may limit independent mobility and feeding, and are linked to cognitive delay, epilepsy, visual, hearing and behavioral difficulties. Developmental disabilities have long term physical, emotional, social, and financial consequences for the child and family in any context, but particularly in low-income country (LIC) settings, where availability of, and access to, support services and inclusive early education are often limited and complicated by financial barriers, social stigma and exclusion (4–8). There is also substantial impact on wider society due to the loss of learning potential and economic productivity, perpetuating poverty in the lowest resource settings (9).

Early programs of care and support have the potential to improve neurodevelopmental outcomes for at-risk children (10). Detecting and intervening early is key to taking advantage of the neuroplasticity of the immature developing brain over the first 3 years of life, to maximize the child’s functioning and developmental potential (11). Importantly, these programs can also have other positive effects on child and family quality of life, health and wellbeing through family capacity strengthening and enrichment of the care-giving environment (12), and optimizing school readiness through promotion of parenting knowledge, skills, and practices (13).

However, programs of early care and support for caregivers of children with developmental disabilities have been under studied, particularly in LIC settings (14, 15). Such programs are wide-ranging in content and approach but may include physiotherapy, occupational, and speech and language therapy interventions, interactive sessions to improve parent-child interactions, and caregiver mental health and peer support, which can be delivered in child development centers, homes or other community locations (14, 16). Several trials have shown positive effects on child motor and cognitive outcomes and caregiver mental health (17, 18) although the populations included were not assessed to have neurodevelopmental impairments (NDIs) and may not all have been particularly “at risk” (19–22). Few studies to date, have examined the feasibility, acceptability, impact and cost-effectiveness of such programs in LICs, and how they might be integrated into existing community programs to promote health and access to early education, although studies are underway (23–28). A systematic, sustained and coordinated approach to implementing and monitoring early detection and intervention initiatives is needed, to improve the life chances of millions of affected children and their families.

In Uganda, it is estimated that 3.5% of all children aged 2–4 years and 7.5% of children aged 5–17 years live with a disability (29); however, only 10% have access to rehabilitative services (15). Empowering mothers to access care and promoting inclusion and participation is key to encouraging early development; in Uganda 51% of women reported full participation in household decision-making, an improvement from 38% in 2011 (30). The Ugandan Ministry of Health have highlighted the need for an integrated policy on early child development, which requires a multisectoral approach comprising health, education, sanitation, empowerment, and safeguarding (31, 32).

**FIGURE 1:**
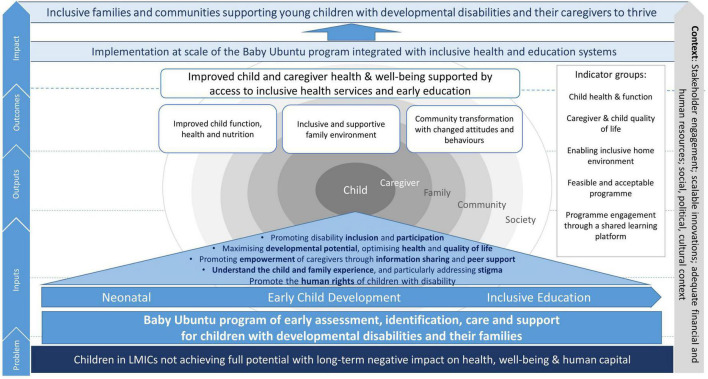
Conceptual framework for the Baby Ubuntu program.

The Baby Ubuntu program is a community-based, group, participatory, peer-led program of early care and support for young children (0–3 years) with developmental disabilities and their caregivers (15, 33), formerly known as the ABAaNA Early Intervention Program.^1^ A conceptual framework of potential pathways to impact of the program at scale is shown in Figure 1. Previous non-controlled pre/post mixed-methods evaluations in Uganda and Rwanda have shown a 15–20% increase in family impact quality of life post-intervention (15, 34), however the feasibility, acceptability, impact and scalability of the program have not previously been formally evaluated. We conducted an individually randomized, pilot feasibility trial of the Baby Ubuntu program, inclusive of a mixed-methods evaluation of (i) program feasibility and acceptability for caregivers and healthcare workers (ii) preliminary evidence of impact when compared with standard care (iii) factors important for scale-up and (iv) provider costs of implementation.

## Materials and methods

A pilot feasibility single-blind, randomized controlled trial with two parallel groups across two study sites, one urban (Kampala) and one rural (Nakaseke), was undertaken. Neither site had existing formal support services for children with developmental disabilities, although referrals to specialist services including pediatric neurology and physiotherapy were possible (Supplementary Figure 1). Full details of the research methodology are described in the published protocol (33).

### Participants and eligibility

Trial participants were infants aged 6–11 months with moderate-severe NDI [defined as a Griffiths Mental Developmental Scales (35) (quotient <70) and/or Hammersmith Infant Neurological Examination (36) (score <60)], and their primary caregivers (most frequently the mother however may be another relative or carer depending on each family’s individual circumstances), and from whom informed written consent was obtained. Exclusion criteria included: age ≥12 months; medical conditions requiring inpatient treatment; unwilling/unable to attend the full program; main residence outside a pre-defined geographic site criterion; non-Luganda or English speakers. Witnessed consent using a thumb print was available to caregivers with low literacy.

### Screening, recruitment and randomization

At-risk infants in the community were screened for eligibility using the Malawi Developmental Assessment Tool (MDAT) (37). Comprehensive neurodevelopmental assessment using the Griffiths Mental Developmental Scales (GMDS) and HINE, was performed for those screening positive for developmental delay. Infants and their caregivers were randomized in a 1:1 ratio to the intervention or standard care (SC) arm using a random number generator prior to the commencement of the study, as previously described (33).

### The intervention

The Baby Ubuntu program manual is freely available to download (footnote 1). The program is divided into ten modules, each lasting 2–3 h, covering understanding disability, positioning and carrying, feeding, mobilizing, communication, play, everyday activities, and the child within the community (34). Modules are delivered over 4–6 months, incorporating at least one home visit. The Baby Ubuntu groups are facilitated by an “Expert Parent,” themselves a caregiver of a child with developmental disability, with or without a healthcare professional. Facilitators receive 5 days of structured training with ongoing supervision and mentorship by a Baby Ubuntu “Master Trainer.” The program manual is freely available to download. Program groups of 6–10 participating families were selected based on locality.

Standard care (SC) referred to existing local services which includes some limited access to physiotherapy and assistive devices, seizure management, audiology, ophthalmology, and nutritional support (Supplementary Figure 1), and this group were offered entry to the program following completion of the study.

### Outcome measurement

#### Quantitative data

Feasibility was evaluated quantitatively as the total number recruited and randomized to each arm over a pre-specified time period (9 months). Acceptability amongst caregivers and healthcare workers was assessed by the protocol violation rate, e.g., participants in the intervention arm being treated as if in the control arm or vice versa, and by pre-specified criteria for “satisfactory attendance” (≥6 modules).

A number of outcome measure tools were piloted and used to examine for early evidence of impact on child and caregiver outcomes (33). These included; *Family quality of life* (QoL) assessed using the scored Pediatric Quality of Life Family Impact module (PedsQL) (38); *Child motor functioning* assessed by the mobility score of the Pediatric Evaluation Disability Inventory (PEDI-CAT) (39); *Child cognitive function* as assessed by the Griffiths Mental Developmental Scales (GMDS) (35); *Child growth and nutritional status* assessed by weight, height, occipito-frontal head circumference and estimation of hemoglobin (HemoCue AB, Angelholm, Sweden); *Caregiver mental health* as assessed using the Self-Referral Questionnaire (SRQ) and the Parenting Stress index (PSI) (40); *Caregiver-child attachment* using the Maternal Infant Responsiveness Instrument (MIRI) (41); and *Quality of the home environment* assessed using the Infant Toddler-Home Observation for the Measurement of the Environment (IT-HOME) (42).

For quantitative data, participants in both arms were assessed by study staff blind to trial allocation, at three time points; pre-intervention (age 6–11 months), at program completion (age 12–17 months), and at 12 months post-completion (age 18–23 months). Outcome measure tools were administered by trained study staff including nurses, doctors, physiotherapist, occupational therapist, medical clinical officer, and a clinical psychologist.

#### Qualitative data

A social scientist conducted in-depth interviews (IDIs) with 20 randomly selected primary female caregivers in the intervention and SC arms at baseline and endline, and nine intervention arm male caregivers at endline. Participants selected for IDI were contacted and interviewed in the local language. Interviews were conducted at the study site, and later transcribed into English by the social scientist for analysis.

To further develop our understanding of program feasibility, acceptability, impact and scale-up, focus group discussions (FGDs) with female caregivers (two FGDs per site) and healthcare workers (one FGD with healthcare workers from both sites) were conducted. In addition, a stakeholder workshop for investigators, study staff, program facilitators and caregivers was held.

### Provider costs

A cost analysis was conducted to examine program costs including set-up (training, equipment and furniture, pre-program expenditure) and running costs (staff, building, supplies, transport refunds, home visits, outreach) over a 1-year time period. Costs relating to the trial, as opposed to implementation of the intervention, were not included in provider costs. Information was gathered from financial data recorded by the project implementation team and facility and program staff interviews. Costs were allocated according to the implementation activities of the program: recruitment, education sessions, and home visits. Costs were inputted into an Excel-based costing tool, in prices in the currency that the cost was incurred; British pounds (GBP) and Ugandan shillings (UGX), and annualized to obtain the economic costs. Costs were incurred in 2018 then inflated to May 2022 prices based on the consumer price index of the currency of the initial recorded cost.^2^ Finally, all costs were converted to 2022 US dollars (USD).

### Sample size

The trial aimed to recruit 126 children and their caregivers; 63 per arm. Allowing for a 20% dropout rate, this sample size gives 90% power to detect a minimal relative difference of 20% on PedsQL Family Impact score between the intervention and control arms, at 5% significance level, assuming a mean PedsQL score of 65 in the SC arm and SD of 20 in both arms (based on data from a pilot pre- and post-evaluation study). The provider cost analysis was performed using all participants completing the program in the intervention arm (*n* = 56).

### Data analysis

Feasibility of participant recruitment and randomization was assessed quantitatively by the total number recruited and randomized to each arm, with feasibility demonstrated if the target sample size of 126 was achieved within the 9-month recruitment period. Acceptability was assessed quantitatively by (i) calculating the protocol violation rate and (ii) summarizing the number of program sessions attended with 6 or more defined as acceptable. Analyses compared outcomes between intervention and control arms at the end of the program, and again 6 months later. On the advice of the DSMB and following CONSORT reporting guidelines for pilot and feasibility trials, we did not plan any formal statistical tests due to the preliminary nature of the trial; instead confidence intervals provide inference around the possible range of effect sizes. Regression models were used to adjust comparisons for baseline measures of the outcomes. Analysis was done on an intention-to-treat basis and missing data were not imputed. The DSMB did not instigate any interim analyses or stopping guidance.

Qualitative data were analyzed using a thematic framework approach around the topics of feasibility, acceptability, impact and scale-up. Two social scientists reviewed the interview transcripts to identify the codes and themes based on the study objectives and other interesting themes that emerged from the data. We described the experiences of children and caregivers relating to the intervention received including the impact of the disability, parental confidence level, inclusion in community life and experiences of stigma and discrimination. We examined changes in these domains over the follow-up period and explored attributions of change. In addition, we performed social mapping of parent networks and group discussions with staff on their perspectives and experiences of using the program.

## Results

**FIGURE 2:**
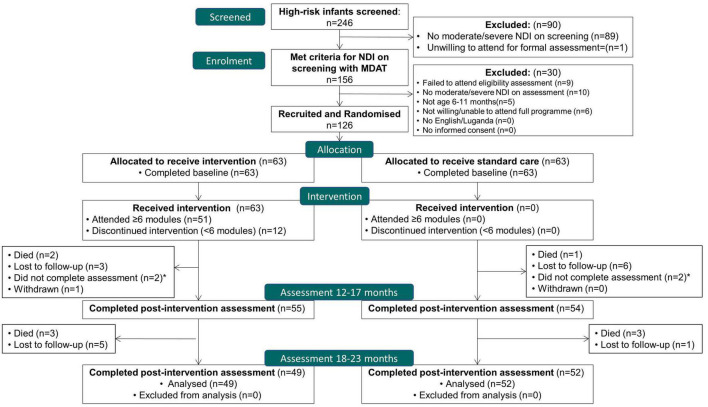
Flow of participants; CONSORT flow diagram. *Two participants in each arm did not complete the 12–17 month assessment but returned for the 18–23 month visit.

In total, 126 infants were recruited between 25th January and 16th October 2018, with 101 (80.2%) participants completing the final follow-up assessment at 18–23 months, by 2nd October 2019. Twelve (9.5%) were lost to follow up, 4 (3.2%) withdrew, and 9 (7.1%) died. Of 63 randomized to the intervention, 59 survived (93%); of these, 51 (86%) attended ≥6 modules, and 49 (83%) completed the follow-up assessment at 18–23 months. The flow of trial participants is outlined in Figure 2.

### Baseline characteristics and descriptive analyses

Baseline characteristics of recruited children are presented in Table 1. Median age at recruitment was 9.5 months. Most recruited infants had severe NDI (GMDS DQ < 55).

**TABLE 1:** Baseline characteristics of study participants by trial arm.

Characteristic	Intervention (*n* = 63)	Standard care (*n* = 63)	Overall (*n* = 126)
Age in months, median (IQR) [range]	9.4 (7.2–10.2) [6.0–11.9]	9.5 (7.8–10.2) [6.0–12.6]	9.5 (7.5–10.2) [6.0–12.6]
Sex (Male)	32 (51%)	32 (51%)	64 (51%)
Mother’s education			
None/Primary	21 (34%)	26 (41%)	47 (38%)
Secondary	29 (48%)	29 (46%)	58 (47%)
Tertiary	11 (18%)	8 (13%)	19 (15%)
Father’s education			
None/Primary	20 (34%)	16 (27%)	36 (30%)
Secondary	22 (37%)	29 (48%)	51 (43%)
Tertiary	17 (29%)	15 (25%)	32 (27%)
DQ median (IQR) [range]	33.4 (16.3–45.5) [0.6–75.5]	27.4 (12.2–36.1) [3.0–80.0]	28.7 (14.2–42.6) [0.6–80.0]
HINE score median (IQR) [range]	33 (23.5–44) [13 –68]	30 (21–47) [10–69]	32.5 (22–46) [10–69]
Weight-for-age z-score, median (IQR) [range]	−2.4 (−3.8, −1.3) [−6.1, 0.8]	−2.4 (−3.3, −1.2) [−5.8, 1.3]	−2.4 (−3.5, −1.2) [−6.1, 1.3]
Head circumference-for-age z-score, median (IQR) [range]	−2.6 (−4.1, −1.1) [−6.0, 6.0]	−2.2 (−3.5, −0.9) [−6.0, 6.0]	−2.4 (−3.8, −1.0) [−6.0, 6.0]

DQ, developmental quotient on griffiths mental development scales II; HINE, hammersmith infant neurological examination.

#### Descriptive analyses of qualitative research participants

Of the 20 randomly selected female caregivers, all participated in IDIs at baseline and 16 (80%) at endline; the four who were not interviewed had a child that died during the study period. Nine of the 20 caregivers (45%) were <25 years old (mean age 25.7 years), and for 55% (11/20) the recruited child was their first born. All but 2 were biological parents; a grandmother and a maternal aunt were caring for two children due to one mother being away and another having a disability. All participants had some primary education and most lived separately from the child’s father (either fully or partially separated) at the time of recruitment. At the rural study site (total 10), the majority (8) were small-scale farmers; at the urban site (total 10) six were engaged in small-scale business.

Male caregivers [total, 9 (5 rural, 4 urban)] interviewed were older (mean 36 years, range 27–52), and all were considered married; 2 in their second marriage, and 3 had more than one wife. All had some primary school education, and 5 had some secondary school education; all were employed, the majority in manual labor.

Focus group discussions at baseline and endline included, 4 with a total of 32 female caregivers (8 from each arm at each site), and 2 FGDs with 10 healthcare workers (4 urban, 6 rural). The stakeholder workshop held in November 2019 was attended by 6 research investigators, 14 study staff or healthcare workers (HCWs), 3 program facilitators, 4 caregivers, and 5 other stakeholders.

### Program feasibility and acceptability

In total, the target number (*n* = 126) of infants were recruited in less than 9 months meeting the primary feasibility outcome. Acceptability of the program was indicated by all children receiving either the intervention or standard care according to allocation (no protocol violations). A total of 51 intervention arm families attended six or more sessions (84%, allowing for the two children who died during the intervention).

Qualitative analysis of the findings provided evidence that the intervention was both feasible and acceptable to most participants, facilitators and HCWs. Major enabling factors cited were peer support from other caregivers; local community members acting as “champions” to support mobilization families to participate in the program; the positive and caring attitudes of HCWs and facilitators toward children with disability creating a conducive enabling social environment; good accessibility of training and materials; and incentives including transport reimbursement. Program barriers included lack of engagement of male caregivers, lack of community awareness around child disability, superstition around etiology of disability including discrimination, and challenges relating to poverty including traveling to sessions. Proposed solutions included active early engagement of male caregivers, earlier and greater emphasis on community sensitization to promote wider engagement and geographically locating groups at more local community clinics rather than central referral hospitals, and the provision of social protection and other livelihood support. Themes and sub-themes emerging from the qualitative analysis on feasibility and acceptability are presented in Supplementary Table 1.

Prior to the program, caregivers and HCWs expressed concerns that particularly in the rural setting, there was very limited provision of care services available for children with disabilities. Whilst specialty services were available at both sites (Supplementary Figure 1), access to these services was complicated by HCWs often lacking knowledge and skills in managing children with disability which hindered communication with caregivers and timely referral. Exclusion of children with developmental disability from services was commonly described by caregivers. One female caregiver reported being sent away from the facility to see a herbalist because she had attended the same facility several times and the child was not improving. This was a theme that came through particularly strongly at the stakeholders meeting.

One female caregiver from the intervention arm shared how she felt when she first joined the program “*I used to ask myself so many questions why my child was different*… *I felt that I was alone, but when (the program coordinator) invited me, she explained to me that we were going to meet in groups and learn how to take care of our children. I got excited*.”

#### Feasibility of the program

Participant recruitment was greatly facilitated through community health outreach by program coordinators and facilitators. HCWs described the importance of community champions in both community sensitization, identification and mobilization of families of children with developmental disability.

Pre-intervention, female caregivers described high levels of stigma and emotional, social and financial burden associated with caring for a child with developmental disability, and the substantial barriers that this represented to program enrollment. Several female caregivers reported that the program helped reduce their own self-stigma and blame, which helped them to understand and accept the condition and situation of their children. An 18-year old female caregiver shared the importance of the program helping her to understand that her child’s disability was not her fault, or caused by “witchcraft,” and that she did not need to segregate him from other children. Community sensitization to issues around child disability were highlighted by both female and male caregivers. Fathers particularly emphasized the importance of sensitizing everyone in the community to the learnings from the program as child disability could affect others; in his words “*Today it’s me, tomorrow it’s you*” (IDI P8).

Amongst barriers to feasibility and acceptability, mentioned by caregivers and HCWs, was the limited engagement of fathers. Whilst none of the interviewed fathers had attended the program directly, most appreciated the support provided to their children and were keen to implement what had been learnt. As one father said: “*as long as you have chosen to be a parent, you have to be involved at all stages, because that is your child too*.” (IDI, P1). Some fathers were exasperated by the number of different programs which come and go at the hospital, especially if they failed to deliver on perceived promises.

“…*I sent in my child with the mother*… *to help out with treating the blisters [on] her head but she was told that they couldn’t help*…*because that was not part of (the program) and she didn’t get any help. So how is the program going to help me?*” (IDI, P4).

At the outset of the study, most caregivers were struggling financially with caring for their child with developmental disability due to increased care needs and reduced ability to work. Some caregivers reported that as a result of participating in the intervention they were able to return to work, having gained skills on how to care for the child. One of the mother’s said, “*I had given up on work since no one could accept to stay with my child, but because I learnt to feed him and how to make him calm, I have returned to work leaving the boy with one of my relatives.*” However, poverty was a clear theme throughout the IDIs. More than half of the female caregivers continued to struggle with transport costs and the cost of specialized care when referred. Caregivers reported poverty as the most important contributor to continued poor quality of life, despite the other positive impacts identified for themselves and their child.

Poverty was commonly identified as a barrier to attendance and was exacerbated by the costs of care, but also loss of income due to caring responsibilities. Prospectively meeting the cost of transport, despite the program reimbursing travel costs, was particularly challenging for some, especially those traveling significant distances due to wide geographical spread of some group members. One female caregiver observed that “*the place was good though it is too far from our homes*.”

Poverty was also cited as a key factor undermining access to care and impact on quality of life. One HCW said “*Most of the families are poor and stay deep in villages so, raising money to take their children for treatment on a regular basis may be difficult*.” Although most caregivers found the training modules especially the practical sessions, easy to implement, some caregivers particularly from rural communities reported that the nutrition module, whilst very useful, was expensive because of the different foodstuffs recommended.

Female caregivers identified the program coordinator as key to success of the groups. They particularly valued the coordinator and facilitators being easy to contact, which enhanced attendance and adherence. Phone call reminders to caregivers about their appointments was valued and reported to positively influence attendance.

#### Acceptability of the program

Facilitators and HCWs were reported to be accommodating, respectful and friendly which made the caregivers feel confident in participating. This strengthened the rapport between HCWs and caregivers, and promoted emotional wellbeing and a sense of acceptance. One female caregiver said, “*I remember on the first day, I came very early in the morning and had not carried tea for my child. She was all crying with mucus dripping from her nose. As soon as I stepped on the door the (healthcare worker) got the baby from me and carried him before I even greeted her*… *it is so rare (and) encouraged me to keep coming*…” The provision of simple refreshments during the group sessions and transport reimbursements were also identified by female caregivers and HCWs as contributing positively to attendance and acceptability.

Most participants within the intervention arm strongly attributed program acceptability, and impact, to the psychosocial support from other caregivers in the group and Expert Parent facilitators. For example, during a post FGD, one of the participants said, “*they could teach us something they have been through which is good*… *and I always wanted to come to meet with them because we would interact freely*.” Group composition of participants from the same or neighboring communities motivated caregivers to attend sessions and promoted peer support from within the immediate community. Peer support through the facilitated group, partnered with individualized support from group facilitators, was reported as particularly powerful in meeting the variable needs of different caregiver-child dyads. Participants reported that community (home) visits supported sharing of individual challenges and barriers in more depth, and provided opportunities for one-to-one discussion of issues.

The use of health facilities as the site for group meetings was well received by participants and many reported that this promoted access to other facility-based health services from which they had frequently felt excluded. In addition, participants reported that group meetings facilitated provision of medication and other adjunct medical and therapeutic consultations, and this further incentivized regular attendance. However, HCWs felt that clearer referral pathways to specialist services were also required, particularly for speech and hearing problems.

Facilitators reported that session debriefs led by Master Trainers were highly valued, enabling reflection on learning experiences, delivery of the program, and enabling them to track progress. The participatory approach of the program promoted caregivers to share their experiences, and encouraged hands-on practice of practical skills such as feeding, improving their confidence in caring for their child. One female caregiver said “*We were taught to communicate with (our children) more often so that their brain may grow and learn how to speak. Putting the child nearby you when you are doing work so that the child can also learn how to do what you doing for example when you are washing utensils*.”

The caregivers and HCWs were positive about the training materials, and the utilization of everyday items. However, some felt financially challenged in needing to procure materials themselves, e.g., foodstuffs for the feeding module. Caregivers were positive about the sessions being delivered in the local language. However, low literacy levels was highlighted as a barrier to feasibility and acceptability by both caregivers and HCWs. They felt that additional visual materials including pictures and videos would be valuable, as well as more translation from English to local languages. Expert parent facilitators reported that the provision of appropriate training materials [“information, education and communication” (IEC) materials] supported effective content delivery; they also strongly valued the ongoing supervision and mentorship offered by the Master Trainers.

Whilst seeing improvement in their child’s functioning encouraged caregivers to return to the program sessions, facilitators and HCWs talked extensively of the need to balance realistic expectations around progress with maintaining hope. This was particularly relevant for those who had children with more severe impairment. Unrealistic caregiver expectations were felt to be a key cause of caregiver disengagement from the program: a female caregiver said, “…*if I do not see any change in my child’s health I stop coming. Because that will be wastage of energy and money for nothing*.”

### Impact of the program

A number of tools for measuring child and caregiver outcomes were piloted to examine for early quantitative evidence of impact. Table 2 reports the crude and adjusted differences seen between the SC and intervention arm immediately and at 6 months post program completion (12 months post-enrollment). Wide confidence intervals consistent with either a beneficial effect, no effect, or a detrimental effect of the intervention were seen (Table 2).

**TABLE 2:** Outcomes at baseline, 6 and 12 months post-enrollment to the baby Ubuntu program, by arm.

Outcome	Baseline measures		Outcomes at 6 months post-enrollment				Outcomes at 12 months post-enrollment			
			
	Standard care (*n* = 63) mean (SD)	Baby Ubuntu (*n* = 63) mean (SD)	Standard care (*n* = 54) mean (SD)	Baby Ubuntu (*n* = 55) mean (SD)	Crude difference (95% CI)^1^	Adjusted difference (95% CI)^2^	Standard care (*n* = 52) mean (SD)	Baby Ubuntu (*n* = 49) mean (SD)	Crude difference (95% CI)^1^	Adjusted difference (95% CI)^2^
**PedsQL**										
Total score	61.2 (19.1)	64.8 (18.7)	60.1 (18.1)	58.2 (18.1)	−1.9 (−8.8, 5.0)	−5.4 (−11.4, 0.6)	58.2 (21.8)	61.5 (22.6)	3.4 (−5.4, 12.1)	−0.7 (−8.9, 7.5)
Physical functioning	65.2 (20.8)	64.8 (21.5)	64.8 (22.0)	61.4 (25.0)	−3.3 (−12.3, 5.7)	−3.9 (−12.1, 4.3)	64.0 (23.2)	64.1 (27.5)	0.1 (−9.9, 10.1)	−1.0 (−10.6, 8.6)
Emotional functioning	53.8 (29.6)	59.4 (27.9)	53.8 (28.8)	55.5 (28.1)	1.7 (−9.2, 12.6)	−0.8 (−10.9, 9.3)	51.4 (30.3)	56.5 (31.4)	5.1 (−7.1, 17.2)	1.1 (−10.3, 12.5)
Social functioning	59.9 (30.2)	63/3 (28.6)	56.0 (31.6)	48.3 (30.3)	−7.8 (−19.6, 4.0)	−10.8 (−22.1, 0.5)	52.2 (34.3)	53.2 (32.7)	1.0 (−12.2, 14.3)	−1.6 (−14.6, 11.5)
Cognitive functioning	77.9 (23.3)	79.0 (24.3)	75.9 (23.9)	71.9 (24.1)	−4.1 (−13.3, 5.1)	−5.7 (−14.0, 2.5)	71.8 (25.1)	78.8 (25.9)	6.9 (−3.1, 17.0)	4.8 (−5.2, 14.9)
Communication	56.7 (27.4)	55.8 (25.5)	48.0 (19.6)	51.2 (23.5)	3.3 (−5.0, 11.5)	2.3 (−5.7, 10.3)	55.1 (22.1)	59.9 (31.8)	4.8 (−6.0, 15.6)	3.0 (−7.6, 13.6)
Worry	44.9 (26.5)	51.9 (26.3)	46.9 (26.0)	44.4 (25.2)	−2.5 (−12.3, 7.3)	−7.0 (−16.0, 2.0)	44.3 (27.9)	51.9 (30.4)	7.6 (−3.9, 19.1)	2.1 (−8.9, 13.1)
Daily activities	54.4 (35.1)	62.8 (33.4)	52.2 (31.6)	51.4 (38.1)	−0.8 (−14.2,12.5)	−7.8 (−20.2, 4.6)	50.5 (35.8)	49.1 (38.0)	−1.3 (−15.8, 13.2)	−4.1 (−18.7, 10.5)
Family relationships	70.7 (28.0)	76.9 (29.3)	74.0 (27.6)	73.7 (29.5)	−0.3 (−11.2, 10.6)	−3.9 (−14.4, 6.6)	69.5 (31.3)	70.9 (32.0)	1.4 (−11.0, 13.9)	−2.4 (−14.2, 9.5)
PEDI mobility score^3^	36.3 (5.9)	35.3 (6.7)	35.7 (7.7)	37.2 (7.5)	1.6 (−1.5, 4.6)	1.5 (−1.3, 4.3)	39.3 (6.8)	39.7 (8.0)	0.5 (−2.6, 3.5)	0.4 (−2.3, 3.2)
**Developmental quotients**										
Global DQ^4^	28.9 (18.5)	31.3 (19.7)	27.6 (19.7)	31.7 (22.3)	4.0 (−4.0, 12.0)	3.0 (−4.5, 10.6)	16.4 (13.9)	20.4 (16.7)	4.0 (−2.1, 10.1)	2.4 (−2.3, 7.0)
Locomotor	28.6 (23.0)	29.8 (23.5)	18.4 (20.4)	21.6 (21.1)	3.2 (−5.1, 11.5)	3.4 (−3.4, 10.3)	16.8 (15.1)	18.6 (18.2)	1.9 (−4.7, 8.5)	1.8 (−3.8, 7.3)
Personal social	33.2 (23.6)	40.2 (27.5)	25.9 (19.2)	30.4 (24.7)	4.5 (−4.2, 13.2)	1.0 (−5.5, 7.5)	20.4 (17.0)	25.1 (19.9)	4.7 (−2.6, 12.1)	2.2 (−3.6, 8.1)
Speech and hearing	36.7 (22.1)	38.3 (23.7)	23.2 (14.6)	29.6 (17.0)	6.4 (0.2, 12.7)	6.1 (0.8, 11.4)	18.9 (12.1)	22.6 (14.0)	3.8 (−1.4, 8.9)	3.1 (−1.3, 7.4)
Eye and hand	21.7 (20.2)	23.9 (21.1)	15.8 (18.8)	21.4 (22.3)	5.6 (−2.6, 13.7)	5.2 (0.2, 10.2)	12.6 (15.4)	17.7 (18.8)	5.2 (−1.6, 12.0)	4.7 (−0.3, 9.7)
Performance	21.4 (17.4)	21.3 (16.0)	15.4 (15.2)	20.5 (20.0)	5.1 (−1.9, 12.1)	6.6 (0.9, 12.3)	13.2 (14.6)	16.6 (16.3)	3.4 (−2.8, 9.5)	4.8 (−0.1, 9.8)
**Anthropometry^5^**										
Weight-for-age z-score	−2.3 (1.5)	−2.5 (1.7)	−2.5 (1.8)	−2.6 (1.7)	−0.1 (−0.8, 0.6)	0.0 (−0.5, 0.5)	−2.8 (1.5)	−3.4 (1.7)	−0.6 (−1.3, 0.0)	−0.5 (−1.0, 0.0)
Height-for-age z-score	−2.0 (1.6)	−2.4 (2.2)	−2.5 (1.8)	−2.9 (1.9)	−0.4 (−1.1, 0.3)	−0.3 (−0.9, 0.3)	−2.8 (1.5)	−3.2 (1.7)	−0.4 (−1.1, 0.2)	−0.3 (−0.9, 0.2)
HC-for-age z-score	−2.2 (2.0)	−2.2 (2.8)	−2.7 (2.5)	−2.5 (2.6)	0.2 (−0.7, 1.2)	0.3 (−0.4, 0.9)	−3.2 (2.3)	−3.0 (2.7)	0.2 (−0.8, 1.2)	0.1 (−0.5, 0.7)
MUAC-for-age z-score	−1.0 (1.4)	−1.1 (1.5)	−1.1 (1.4)	−1.0 (1.9)	0.1 (−0.6, 0.8)	0.3 (−0.2, 0.9)	−1.1 (1.2)	−1.6 (1.8)	−0.5 (−1.1, 0.1)	−0.4 (−0.9, 0.2)
**Caregiver wellbeing**										
SRQ score^6^	7.0 (4.7)	6.9 (4.7)	7.3 (4.9)	7.9 (5.2)	0.6 (−1.4, 2.6)	1.1 (−0.7, 3.0)	8.5 (5.2)	7.5 (4.9)	−1.0 (−3.0, 1.0)	−0.5 (−2.3, 1.3)
MIRI score^7^	77.3 (12.7)	78.8 (13.6)	80.5 (12.7)	81.6 (12.0)	1.1 (−4.1, 6.3)	−0.3 (−5.4, 4.7)	82.2 (14.3)	84.4 (12.7)	2.2 (−3.3, 7.7)	0.4 (−4.7, 5.5)
PSI score^8^	90.2 (28.0)	90.9 (26.1)	91.2 (26.4)	87.6 (24.8)	−3.6 (−13.7, 6.4)	−6.8 (−15.9, 1.4)	84.6 (29.2)	91.7 (27.6)	7.2 (−4.8, 19.1)	2.8 (−7.3, 12.8)
**HOME^9^**										
Total score	21.8 (4.5)	20.0 (5.0)					24.7 (4.8)	22.2 (5.1)	−2.5 (−5.4, 0.5)	−1.2 (−3.7, 1.3)
Responsivity	5.8 (2.1)	5.3 (2.6)					7.0 (2.1)	6.8 (2.2)	−0.1 (−1.4, 1.1)	−0.0 (−1.3, 1.2)
Acceptance	5.3 (1.1)	5.5 (0.8)					5.5 (0.9)	5.5 (0.7)	0.0 (−0.5, 0.5)	0.0 (−0.5, 0.5)
Organization	3.6 (1.1)	3.9 (1.1)					4.6 (1.0)	3.7 (1.3)	−0.9 (−1.6, −0.2)	−1.0 (−1.6, −0.4)
Learning material	2.8 (2.2)	1.7 (1.6)					2.6 (2.2)	1.7 (1.3)	−0.9 (−1.9, 0.2)	−0.3 (−1.3, 0.7)
Involvement	2.9 (1.1)	2.7 (1.2)					3.3 (1.3)	3.2 (1.2)	−0.1 (−0.9, 0.6)	0.0 (−0.7, 0.7)
Variety	1.3 (1.4)	0.9 (0.9)					1.7 (1.0)	1.3 (1.0)	−0.4 (−1.0, 0.2)	−0.3 (−0.9, 0.3)

PEDI, pediatric evaluation of disability inventory; HC, head circumference; MUAC, mid-upper arm circumference; SRQ, self-report questionnaire; MIRI, maternal-infant responsiveness index; PSI, parent stress index; HOME, home observation for the measurement of the environment. ^1^Baby Ubuntu vs. standard care (reference group). ^2^Adjusted for corresponding outcome assessed at baseline (before randomization). ^3^PEDI missing for 5 in standard care arm, 6 in Baby Ubuntu arm at 6 months and missing for 4 in standard care arm, 2 in Baby Ubuntu arm at 12 months. ^4^Griffiths sub-quotient scores missing for 3 in standard care arm, 2 in Baby Ubuntu arm at baseline and missing for 4 in standard care arm and 4 in Baby Ubuntu arm at 6 months. ^5^Anthropometry missing for 1 in Baby Ubuntu arm at 12 months, z-scores below −6 were imputed to have value −6. ^6^SRQ missing for 5 in standard care arm, 7 in Baby Ubuntu arm at 6 months and missing for 1 in standard care arm at 12 months. ^7^MIRI missing for 9 in standard care, 11 in Baby Ubuntu arm at 6 months and missing for 3 in standard care arm, 3 in Baby Ubuntu arm at 12 months. ^8^PSI missing for 5 in standard care arm, 2 in Baby Ubuntu arm at 6 months and missing for 4 in standard care arm, 7 in Baby Ubuntu arm at 12 months. ^9^HOME done for 27 at baseline and 24 at 12 months in standard care, done for 23 at baseline and 22 at 12 months in Baby Ubuntu arm, not done at 6 months.

Whilst quantitative tools did not provide clear evidence of program impact, qualitative findings provided supportive evidence of impact for children, caregivers and healthcare workers. Key reported impacts included improved perceptions and attitudes toward the ability of children with disabilities, caregiver psychosocial and emotional wellbeing, child function and wellbeing, confidence in child care, peer-support and information sharing. Themes and sub-themes from the qualitative analysis on impact are presented in Supplementary Table 1.

#### Impact for children

The most reported positive impact for children, particularly in the urban site, was greater inclusion in everyday life at both family and community level. Prior to the program, caregivers reported societal exclusion, for example, being cast out by their families, children denied clan names, and HCWs refusing to treat children. Following the program, caregivers reported being encouraged by their family to take their child to hospital, HCWs explaining their treatment plan, and being visited by and invited to eat with members of the community. One of the mothers explained that when she learnt the importance of play and peers during a group session, she approached her neighbors, explained her son’s condition and invited their children to play with her son. This improved her son’s social skills and facilitated his acceptance by community members. Another mother said: “*When my child goes to play at the neighbors, they no longer chase him [after they] explained to them the causes of the condition*.”

Most female caregivers receiving the program reported improvements in their child’s health, development and function. One mother of a child with hydrocephalus said, “*Whenever you are trying to play with him, he extends the hand to you, he is able to turn himself and lie on his stomach, when you try to cover him from direct sunlight, he pulls off the cloth, he eats very well, he is able to sit when I help him lift his head*… *he is ever happy*.”

Similarly, 7 of the 9 fathers interviewed reported seeing a positive impact of the program on their child, including their child’s growth, energy levels, and motor and language development: “*Now you can see that what she was unable to do in the past, she can now do*.” However, two of the fathers reported seeing no impact; one said “*I have not seen any difference or anything she has gotten from the program maybe apart from the transport reimbursement and the questions she is asked whenever she comes to the hospital for a review*.”

In the intervention group, caregivers were supported in accessing routine child health services, and were referred to relevant specialist services. Due to the care and knowledge shared, HCWs reported that children avoided recurring health issues that may have led to secondary disabilities. Caregivers reported that they were better able to identify when their child had health issues and when to seek care appropriately: “*My child does not talk but from what I was taught, I know when he is hungry, sick or about to get sick and I don’t wait*… *I either call (the Baby Ubuntu coordinator) or go to our clinic*.”

#### Impact on caregivers

Most caregivers reported positive impacts on their psychosocial and physical wellbeing, peer support and advocacy. Many reported feeling “love” and “hope” for the first time since their child’s disability had become apparent. For many, however, there was a diminishing of perceived positive effects over time, particularly in emotional wellbeing.

Prior to the program, caregivers reported negative emotions and physical symptoms such as anger, fatigue, and headaches. For example, one of the rural site female caregivers said, “*My child cries day and night. I find it difficult to concentrate and do some work because every time I am carrying the child*… *I feel like abandoning it to his family because even his father stopped supporting us*…(she broke down into tears and as she cleaned her face, she added), *I don’t know what to do*…” Caregiver attitudes toward disability in the intervention arm changed and they became more resilient and hopeful with reported reductions in stress, isolation, and self-stigma. After the program, each of the caregivers reported a positive change in their own psychosocial wellbeing. They reported that the program increased their understanding of their child’s condition and facilitated acceptance of their child, restoring hope and happiness.

Benefits identified by caregivers included a clear understanding of their child’s condition. Witnessing an improvement in their child’s development gave them hope and encouraged caregivers to return to the groups; though conversely a lack of developmental progression left some caregivers disheartened.

Caregivers reported becoming increasingly confident in caring for their children and becoming “child disability champions.” They particularly valued the peer support aspects of the program including sharing information and supporting one another, as well as other families with affected children not participating in the study. One of the mothers said:

“*I preach the gospel everywhere I go, taxis, markets, shops, etc. because I was taught. I tell them that ‘omwana yakoowa’ (child born with birth asphyxia), I tell people a lot about the condition if they ask. I also normally tell people with such children to take them for physiotherapy. I have got many friends through this and I am no longer despised because of my child’s condition. People keep coming to me, especially young girls who are pregnant, and I also advise them to go to hospital early. I no longer feel ashamed*.”

Female caregivers from the control arm mentioned that although they had a place to take their children for treatment, they did not receive much help, and this was evident from their emotional states during interviews. However, through social networks within communities, a few of the control arm mothers had been linked to rehabilitation centers and thereafter had noticed some positive changes in their children.

#### Impact on healthcare workers

Healthcare workers reported positive changes in their perception of child disability and ability to support affected children and families; this increased their hope and motivation. They reported improvement in the quality of their service delivery due to enhanced skills and knowledge, including a better understanding of referral pathways and resources. This contrasted with prior to the program, particularly in the rural setting, a lack of understanding about child disability reported by HCWs which negatively affected their clinical management, communication with caregivers, and timely referral to specialist services. One HCW said, “*Children at risk are now given timely and proper care unlike before*…” Another stated, “*Our attitude, and that of the caregivers toward these babies, has changed. We no longer view them as useless babies because we have seen most of them achieve*…”

Awareness of the etiology also increased; a midwife said in a FGD, “*Being a midwife, I am very keen now and supportive to mothers during labor. I knew the effects of asphyxia even before this intervention, but knowing did not call for any action. Being part of this program has provoked me to take action and I have made initiatives to talk to my fellow midwives about the dangers of asphyxia and how it can be prevented.*” HCWs also appreciated that treating children with disabilities requires collaboration within the multi-disciplinary team, promoting teamwork and a sense of working together to achieve common goals. However, some of the HCWs reported that the increased referrals from the community led to a marked increase in their workload. Whilst this in part related directly to clinical care, they also found that in the absence of social workers it became their responsibility to provide psychosocial support to caregivers which substantially increased their burden.

#### Impact on wider family and community

Prior to the program, all caregiver participants reported that they received little or no support, and they attributed this to stigma around child disability and high levels of discrimination. Post program, female caregivers reported receiving increased social support from medical teams, community members and families. They frequently attributed this to increased community awareness around child disability facilitating support systems at community level. Caregivers described the program as demystifying community superstitions that their child’s condition was a curse or contagious; one female caregiver said “*Ever since my husband interacted with the (program team) my relationship with him improved. He even asks me whether I have done certain roles like feeding him*…*it seems he knows what to do now*.”

However, there were still reports of low levels of support from some extended family members, and continued stigma particularly amongst the caregivers whose child’s progress was slower. Whilst female caregivers were generally positive regarding the impact on the wider family and community, none of the fathers interviewed mentioned this.

### Scalability

Themes and sub-themes from qualitative data analysis on scale-up are shown in Supplementary Table 1.

Facilitators to scale-up of the program were identified during interviews with caregivers and during the stakeholders’ workshop. The key facilitator mentioned by most of the participants was the relevance of the program to potential users. They reported that the program was suitable for their needs and the demand for similar services in communities was high. However, several mentioned the importance of increasing awareness in the community through community meeting and radio/television programs, in addition to peer-to-peer communication. One female caregiver said“…*teaching about this condition on television and radio (is important) because there are people still hiding their children away in the house not wanting others to see them*…*and it is difficult to sensitize her about the child’s condition and the available solution*.” Community engagement was seen as key and a strong driver to successful scale-up. Caregivers mentioned that engaging fathers, religious leaders and traditional healers as advocates influenced acceptability and therefore scale-up. They gave examples of the strong existing beliefs and authority of these community members, and the importance of collaboration to enable access.

“*When I had just got this child, I was advised to go to different powerful people. I went to priests, pastors, witch doctors and old women and they had their own explanations. The pastors were telling me it was a curse, the witch doctors and elders were telling me it was something to do with clan spirits and though all of them gave me what to use, none of it worked until I came here*.”–Female Caregiver

On the same note, HCWs gave examples of existing service providers and disabled people’s groups to collaborate with for capacity building and service delivery, to improve cost-effectiveness and sustainability. Bringing services closer to people was felt to increase access, use and involvement. Most families were unable to access intervention services due to distance, and HCWs’ limited understanding of the needs of families at the community level. They felt that integrated community services would increase fathers’ engagement, acceptance, peer-to-peer support and timely intervention seeking.

“…. *if the program can be taken out of the hospitals to the communities just like it is for HIV. This is because most of the people in communities don’t know that our children are in this condition because they were born tired, they think it is due to supernatural powers like witchcrafts, sacred oracles and so keep demoralizing us and our husbands from giving us support*.”–Female caregiver

Poverty of participants and lack of finances for transport also limited attendance. One female caregiver said, “*Most of the families are poor and stay deep in villages so, raising money to take their children for treatment on a regular basis may be difficult*.” Limited resources and capacity of healthcare services, with rigid systems/teams and lack of political were also considered important barriers to scale.

Government financial and political investment, at local, regional and national levels, was identified as important for scalability and sustainability at the stakeholder workshop. Furthermore, stakeholders felt that involvement of non-governmental organizations operating in the region could help roll-out and scale-up of the program. Stakeholders also mentioned development of a “train the trainers” program as key in facilitating scale-up of Baby Ubuntu master trainers.

### Costs of the program

The total costs of setting up and running the program from a provider perspective were USD 14,732.26. The running costs of the program per participant were $232.01 if transport reimbursement for participants was included, and $160.90 if excluded. The total setup cost for the program was $1,739.62, equivalent to a per participant setup cost of $31.06 (Table 3).

**TABLE 3:** Program provider economic costs in 2022 US Dollars (USD) (*n* = 56).

Cost category	Type of cost in 2022 USD		
	
	Cost	% of Total cost	Cost per participant
**Set-up costs**			
Equipment and furniture	511.15	29.38%	9.13
Training and pre-trial expenses^1^	1,228.48	70.62%	21.94
Total set-up costs	**1,739.62**	**11.81%**	**31.06**
**Running costs**			
Staff^2^	3,971.67	30.57%	70.92
Building costs^3^	2,426.80	18.68%	43.34
Refreshments	416.24	2.83%	7.43
Office supplies	346.46	2.35%	6.19
Airtime	740.23	5.02%	13.22
Transport refund for participants	3,982.02	30.65%	71.11
Transport costs for facilitators	79.67	0.61%	1.42
Home visits^4^	1,029.55	7.92%	18.38
Total running costs	**12,992.64**	**88.19%**	**232.01**
Total cost	**14,732.26**		**263.07**

^1^Includes costs of formatting and printing the education guides, toys and mats for children, and training of implementors. Costs of development and piloting of education guide were excluded. ^2^Includes proportion of gross salary (inclusive of net pay + National Social Security Fund + Pay as You Earn) multiplied by time allocated to project implementation. ^3^The cost of the square footage of hospital spaces used for study activities as a fraction of the total facility cost multiplied by the number of hours of use for education sessions. ^4^Inclusive of both staff time and transport costs, as these were compensated at a fixed rate per participant.

## Discussion

Baby Ubuntu is a community-based, participatory group rehabilitation program, co-facilitated by healthcare workers and expert parents, that aims to provide an affordable solution to providing early care and support for young children with developmental disability and their caregivers and promote access to child health services and early education. In this pilot feasibility trial in Uganda, with high levels of identified need, Baby Ubuntu was found to be feasible and acceptable in both urban and rural settings. Whilst our mixed methods evaluation provided qualitative evidence of impact on family knowledge, skills, and attitudes, quantitative evaluation of family impact quality of life was inconclusive amongst this population of children with severe developmental impairments. Important programmatic barriers included stigma and exclusion, poverty, and the need to manage expectations around the child’s progress. Facilitating factors for scale included community level engagement and sensitization around child disability, and the need to embed the program within existing community health systems. Limited capacity of already overstretched existing healthcare systems was also identified as a challenge. The provider cost estimate represents a feasible intervention for this vulnerable group, encouraging financial sustainability at scale.

Quantitative and qualitative findings supported the program’s feasibility and acceptability to most participants, facilitators and healthcare workers. Important enablers identified were peer-support from fellow caregivers, and respectful care toward children and their caregivers by program facilitators, which strongly facilitated attendance. Participatory content was reported to support caregivers in understanding their child’s lived experience and needs, relevant not only to meeting immediate care needs, but also accessing routine child health services and early education from which they had been frequently turned away (13, 43, 44). In our study, high levels of self- and community-level stigma were experienced by caregivers, in accordance with our previous research (4, 45, 46), presenting a substantial barrier to accessing services, including healthcare, rehabilitative services and early education. This highlights the need for interventions to consider community-level enablers and barriers and to broaden the programmatic focus from the child to include the wider family and community. Specifically community awareness around childhood disability was seen as crucial in combatting superstition, discrimination and exclusion (47). Early identification of eligible families was strongly facilitated by local community champions, with stakeholders clearly identifying the need for integration with current established community healthcare systems to support early identification of those most in need.

Barriers to attendance included poverty, geographical challenges (distance traveled) and lack of engagement of male caregivers. The effects of poverty on a child’s development and education readiness has been found to be highest in the earliest years, mediated through several factors including the home environment (13). Given the strong influence of the home on young children’s learning and development, the lack of ability of low-income families to modify the effects of poverty may inhibit access to both healthcare and early education. Traveling long-distances to meet with their group was stated as a barrier by many, despite provided transport reimbursements. Increasing program reach and coverage would likely mitigate this by reducing the geographical coverage of individual groups. Whilst there is a tendency for health and education programs to focus on supporting female caregivers, who frequently shoulder the greatest caregiving responsibility, paternal engagement is clearly key and has been associated with a number of positive development and education outcomes (13). In our study, fathers were frequently considered gatekeepers of access to groups, health services and, by extension, early education.

Qualitative findings reported positive impacts on a range of child and caregiver outcomes, including perception of the child’s health and abilities, however this did not clearly translate to quantitative measures of neurocognitive functioning and quality of life. Whilst intensive therapy interventions for infants with disabilities have been shown to have positive impact on early child functioning (22, 48), it is recognized that this is more challenging in those with the severest of NDIs as seen in the Baby Ubuntu trial cohort. Tools for measuring outcomes lack standardization and validation in LMICs (14), and may not have been sensitive or specific enough to detect a change in our cohort. Positive qualitative experiences reported by caregivers may reflect psychological bias from the belief in the intervention, peer support and motivation. It is possible that the early identification of children at a young age, when the extent of their developmental disability is just becoming apparent, may also undermine the ability for impact on quality of life as families come to terms with their child’s condition. Poverty was also a commonly identified barrier to both feasibility and impact, meaning that whilst improvements in knowledge, skills and attitudes were valued, they did not always translate to an improvement in quality of life at family level when poverty still remained. It is also possible, that a longer period of administration (dose dependence) is required to have a positive and holistic impact on the child, caregiver and family. These may explain the significant improvement in family quality of life seen in pre- and post-evaluations, in previous non-controlled studies (15, 34).

The socio-emotional and psychological impact of caring for a child with early child developmental disability on caregivers was clearly reported. Many female caregivers showed physical signs of psychosocial distress during baseline interviews however described the program as transformational in their attitude and behaviors, leading to a more accepting and loving parenting approach. The peer support of others, themselves caregivers of children with disability, was described as key in reducing feelings of isolation, suggesting the important contribution of this to both acceptability and impact. The environment provided in the home, indicated by caregiver engagement in learning activities such as play, is considered to be a strong characteristic of developmental and educational readiness of families (13).

With regards to factors important for scale-up, stakeholders reported a high level of need and demand in the community for early care and support parenting interventions. Community sensitization and engagements were identified as crucial in successful implementation and impact of the program. Stakeholders reported community acceptability attributed to the relevance of the intervention, and of the participatory approach of delivery. In particular, this enabled acceptability and buy-in that can be leveraged to enable both scale-up and sustainability. Participatory approaches have been demonstrated elsewhere to promote smooth integration and scale up of interventions in public health and education (49). Community engagement promoted ownership, support and advocacy for affected families, and enabled smooth implementation, but also highlighted the need to embed the program within existing community health systems. The main barrier to implementation at scale identified by stakeholders, was the limited capacity of already overstretched existing healthcare systems. This has been identified previously in an evaluation of human resources across several early child development programs (50).

The reported provider costs associated with delivering the program add to the evidence of feasibility. The majority of costs were running costs, largely related to workforce and transportation reimbursement for participants. In this research study, transportation costs were largely provider allocated, however this would be less likely if integrated with community health services at scale. Increased program reach and coverage should however reduce this cost through closer geographically located groups. In addition, there may be additional economies at scale, when implementation is streamlined and potentially more efficient (51), particularly when integrated into routine community health services (52). Overall, there is a relative paucity of costing data on parenting interventions for children with disabilities in LIC settings. One cost-effectiveness analysis of a community-level intervention for children and adults with disabilities in Nepal reported a cost per participant of 630 Euros (53). However, this study included both adults and children with a wide range of disabilities and followed a community-based rehabilitation model including health, education, livelihoods, social context and empowerment, and thus may not be directly comparable.

### Strengths and limitations

Our Baby Ubuntu feasibility trial reports some of the first evidence from sub-Saharan Africa on feasibility and acceptability of parenting interventions for young children with developmental disabilities. Whilst a comprehensive systematic review of parenting interventions for children during the first 3 years of life supported impact on early child development outcomes across all income settings, programs targeting children with developmental disabilities were not included (54). Given that the trial evaluated a participatory, facilitated group intervention, it was not possible for program facilitators and all research staff to be blind to allocation. However, to mitigate any bias in reporting outcome measures, study staff performing endline assessments were blind to allocation arm and all other clinical data, with staff from the urban site conducting the rural site assessments and vice versa. It was challenging to protect against the community-level component of the intervention contaminating the control arm in this individually randomized trial, and there were known incidences that intervention arm caregivers shared program content with those from the control arm. It is possible that this undermined the ability to observe differences in outcomes between the arms. A cluster-randomized designed trial is planned for more rigorous evaluation of process, impact, and cost-effectiveness of this complex intervention. It is also possible that the outcome measure tools themselves were limited in their ability to detect change through lack of validation in the LIC setting or through difficulties with accurate translation and interpretation. All attempts were made to mitigate this risk, including choosing validated tools where possible, and those that have been used previously in LIC studies, ideally studies relating to child disability.

## Conclusion

The Baby Ubuntu program aims to provide an affordable solution to early care and support for children with developmental disability and their families. Our feasibility trial found the Baby Ubuntu program to be feasible and acceptable to families in both urban and rural settings in Uganda. This mixed methods evaluation provided strong qualitative evidence of impact on family knowledge, skills, attitudes and quality of life, however this was less clear on quantitative evaluation. Facilitating factors for scale included community level engagement and sensitization around child disability and the need to embed the program within existing community health systems. Important barriers included stigma, poverty, limited capacity of existing healthcare systems and highlighted the need to manage expectations around the child’s progress. The cost estimate represents a feasible intervention for this vulnerable group, encouraging financial sustainability at scale. Planned work includes integration of Baby Ubuntu within government community health systems in Uganda and Rwanda, development of program modules targeting pre-school readiness and livelihood support, and a cluster-randomized trial and mixed-methods evaluation of process, impact and cost-effectiveness at scale.

## Data availability statement

The raw data supporting the conclusions of this article will be made available by the authors, without undue reservation.

## Ethics statement

The studies involving human participants were reviewed and approved by London School of Hygiene & Tropical Medicine; MRC/UVRI and LSHTM Uganda Research Unit; Mulago Hospital; Kiwoko Hospital; Uganda National Council for Science and Technology; Uganda President’s Office. Written informed consent to participate in this study was provided by the participants’ legal guardian/next of kin.

## Author contributions

CT conceived and designed the feasibility trial with substantial contribution from MN, DL, JN, EW, CM, JS, KK, and FC. MN, CN, MZ, JN, BM, DK, SS, RN, FC, AM, and CT developed trial methodology. CN, MK-L, SS, RN, JN, AM, KK, CO, and MN conducted the data collection. EW, RN, and CT conducted the data analysis. KK and GG designed the cost analysis. KK conducted data collection. ET and KK conducted data analysis under the guidance of GG. CT, MN, SS, MK-L, CN and ET were written the first version of the manuscript. All authors contributed to the final version of the manuscript.

## Footnotes

1 Baby Ubuntu early intervention program online resources: https://www.ubuntu-hub.org/resources/babyubuntu/.

2 Uganda consumer price index 2016/17: https://www.ubos.org/wp-content/uploads/publications/12_2021CPI_PUBLICATION_NOVEMBER_2021.pdf.

## Supplementary material

Supplementary Figure 1 Standard care referral pathways for children with developmental concerns/disability at Mulago Hospital (urban site) and Kiwoko Hospital (rural site).
