# Supplementary material for: Early care and support for young children with developmental disabilities and their caregivers in Uganda: The Baby Ubuntu feasibility trial
Source: Front Pediatr. 2022 Sep 13;10:981976. doi: 10.3389/fped.2022.981976 (PMC9513138; doi:10.3389/fped.2022.981976)
Supplement: Supplementary file 1 [file Table_1.docx]

**Supplementary Material Table 1: Themes and sub-themes from the qualitative analysis on feasibility, acceptability, impact and scale-up**

|  | THEMES | SUB-THEMES |
| --- | --- | --- |
| Feasibility | 1. Gap in services for children with disabilities | 1.1 Need for the intervention |
|  | 2. Community sensitisation & family engagement | 2.1 Role of local community champions |
|  |  | 2.2 Stigma and discrimination |
|  |  | 2.3 Paternal engagement |
|  | 3. Financial challenges | 3.1 Poverty |
|  | 4. Program management | 4.1 Coordination of sessions |
| Acceptability | 5. Enabling social environment & psychosocial support | 5.1 Positive and caring attitudes of HCWs |
|  |  | 5.2 Peer support |
|  | 6. Programme delivery | 6.1 Combination of groups vs individual sessions |
|  |  | 6.2 Facilitator training & supervision |
|  |  | 6.3 Access to other services |
|  |  | 6.4 Incentives and facilitation |
|  | 7. Programme content | 7.1 Participatory approach to learning |
|  |  | 7.2 Improved confidence in knowledge and skills |
|  |  | 7.3 Accessibility of program materials & equipment |
|  | 8. Managing expectations | 8.1 Caregivers’ expectations around progress |
| Impact | 9. For children | 9.1 Social inclusion |
|  |  | 9.2 Health, well-being, function & nutrition |
|  | 10. For caregivers | 10.1 Psychosocial and physical well-being |
|  |  | 10.2 Peer support and advocacy |
|  |  | 10.3 Financial barriers |
|  | 11. For healthcare workers | 11.1 Perceptions and attitudes to child disability |
|  |  | 11.2 Multi-disciplinary working and referral |
|  |  | 11.3 Workload & capacity |
|  | 12. For wider family and community | 12.1 Support with caring |
|  |  | 12.2 Community sensitisation to the needs of children with disabilities |
| Scale-up | 12. Integration with community health systems | 12.1 Community & stakeholder engagement |
|  |  | 12.2 Integrating identification with intervention |
|  | 13. Human resources | 13.1 Capacity building |
|  |  | 13.2 Workload |
|  |  | 13.3 Training, supervision & fidelity |
|  | 14. Programme fidelity | 14.1 Standardisation of content vs. adaptability to context |
|  |  | 14.2 Monitoring, evaluation & learning |
|  | 15. Cost of the intervention | 15.1 Cost implications |
