# Supplementary figures and images for: Early care and support for young children with developmental disabilities and their caregivers in Uganda: The Baby Ubuntu feasibility trial
Source: Front Pediatr. 2022 Sep 13;10:981976. doi: 10.3389/fped.2022.981976 (PMC9513138; doi:10.3389/fped.2022.981976)

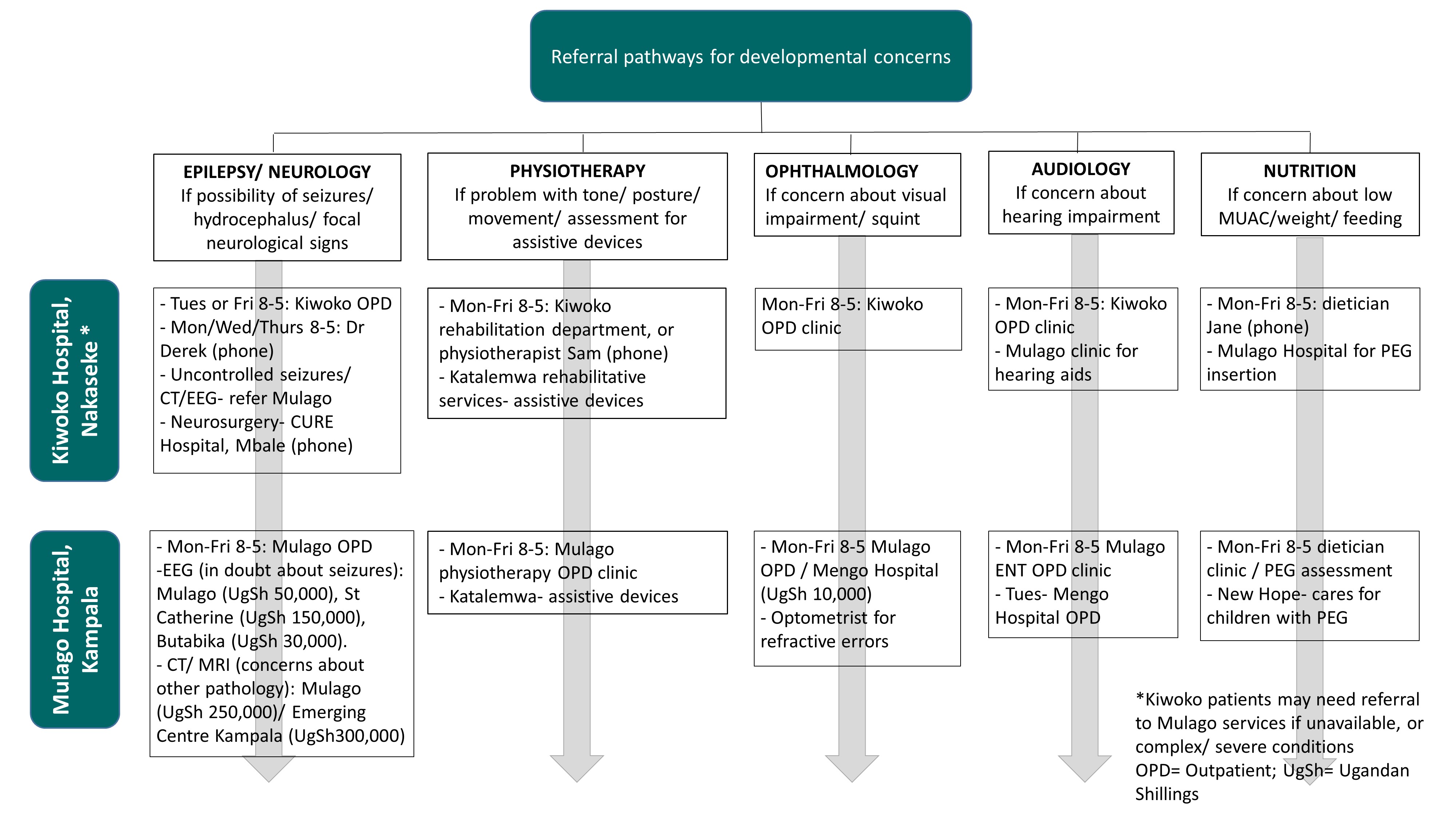

Supplement: Supplementary Figure 1 — Standard care referral pathways for children with developmental concerns/disability at Mulago Hospital (urban site) and Kiwoko Hospital (rural site). [file Image_1.JPEG]
